# Setting the scene and generating evidence for malaria elimination in Southern Mozambique

**DOI:** 10.1186/s12936-019-2832-9

**Published:** 2019-06-06

**Authors:** Pedro Aide, Baltazar Candrinho, Beatriz Galatas, Khátia Munguambe, Caterina Guinovart, Fabião Luis, Alfredo Mayor, Krijn Paaijmans, Lucía Fernández-Montoya, Laia Cirera, Quique Bassat, Sonia Mocumbi, Clara Menéndez, Delino Nhalungo, Ariel Nhacolo, Regina Rabinovich, Eusébio Macete, Pedro Alonso, Francisco Saúte

**Affiliations:** 1 Centro de Investigação em Saúde de Manhiça (CISM), Manhiça, Mozambique; 2 National Institute of Health, Ministry of Health, Maputo, Mozambique; 3 National Malaria Control Programme, Ministry of Health, Maputo, Mozambique; 4 ISGlobal, Hospital Clínic - Universitat de Barcelona, Barcelona, Spain; 5 School of Life Sciences, Center for Evolution and Medicine, Biodesign Center for Immunotherapy, Vaccines and Virotherapy, Arizona State University, Tempe, USA; 6 ICREA, Pg. Lluís Companys 23, 08010 Barcelona, Spain; 7 CIBER Epidemiología y Salud Pública (CIBERESP), Madrid, Spain; 8 National Directorate of Health, Ministry of Health, Maputo, Mozambique; 9 Universidade de Eduardo Mondlane, Maputo, Mozambique; 10 Harvard T.H. Chan School of Public Health, Boston, MA USA

**Keywords:** Malaria, Elimination, Mozambique, Alliance, Magude project

## Abstract

Mozambique has historically been one of the countries with the highest malaria burden in the world. Starting in the 1960s, malaria control efforts were intensified in the southern region of the country, especially in Maputo city and Maputo province, to aid regional initiatives aimed to eliminate malaria in South Africa and eSwatini. Despite significant reductions in malaria prevalence, elimination was never achieved. Following the World Health Organization’s renewed vision of a malaria-free-world, and considering the achievements from the past, the Mozambican National Malaria Control Programme (NMCP) embarked on the development and implementation of a strategic plan to accelerate from malaria control to malaria elimination in southern Mozambique. An initial partnership, supported by the Bill and Melinda Gates Foundation and the La Caixa Foundation, led to the creation of the Mozambican Alliance Towards the Elimination of Malaria (MALTEM) and the Malaria Technical and Advisory Committee (MTAC) to promote national ownership and partner coordination to work towards the goal of malaria elimination in local and cross-border initiatives. Surveillance systems to generate epidemiological and entomological intelligence to inform the malaria control strategies were strengthened, and an impact and feasibility assessment of various interventions aimed to interrupt malaria transmission were conducted in Magude district (Maputo Province) through the “*Magude Project*”. The primary aim of this project was to generate evidence to inform malaria elimination strategies for southern Mozambique. The goal of malaria elimination in areas of low transmission intensity is now included in the national malaria strategic plan for 2017–22 and the NMCP and its partners have started to work towards this goal while evidence continues to be generated to move the national elimination agenda forward.

## Background

In 2014 the Mozambican National Malaria Control Programme (NMCP) and its partners started to work towards the global vision of a malaria-free world. This vision materialized in the NMCP’s strategic plan for 2017–2022, which focuses on burden reduction in high endemic areas while sustaining the gains in low transmission areas to accelerate towards elimination. This article reviews the historical malaria control and elimination approach in Mozambique, and details the malaria elimination activities that the country has embarked on since 2014 in order to develop and implement a malaria elimination plan for southern Mozambique.

## The history of malaria control in Maputo Province, Southern Mozambique

**Fig. 1:**
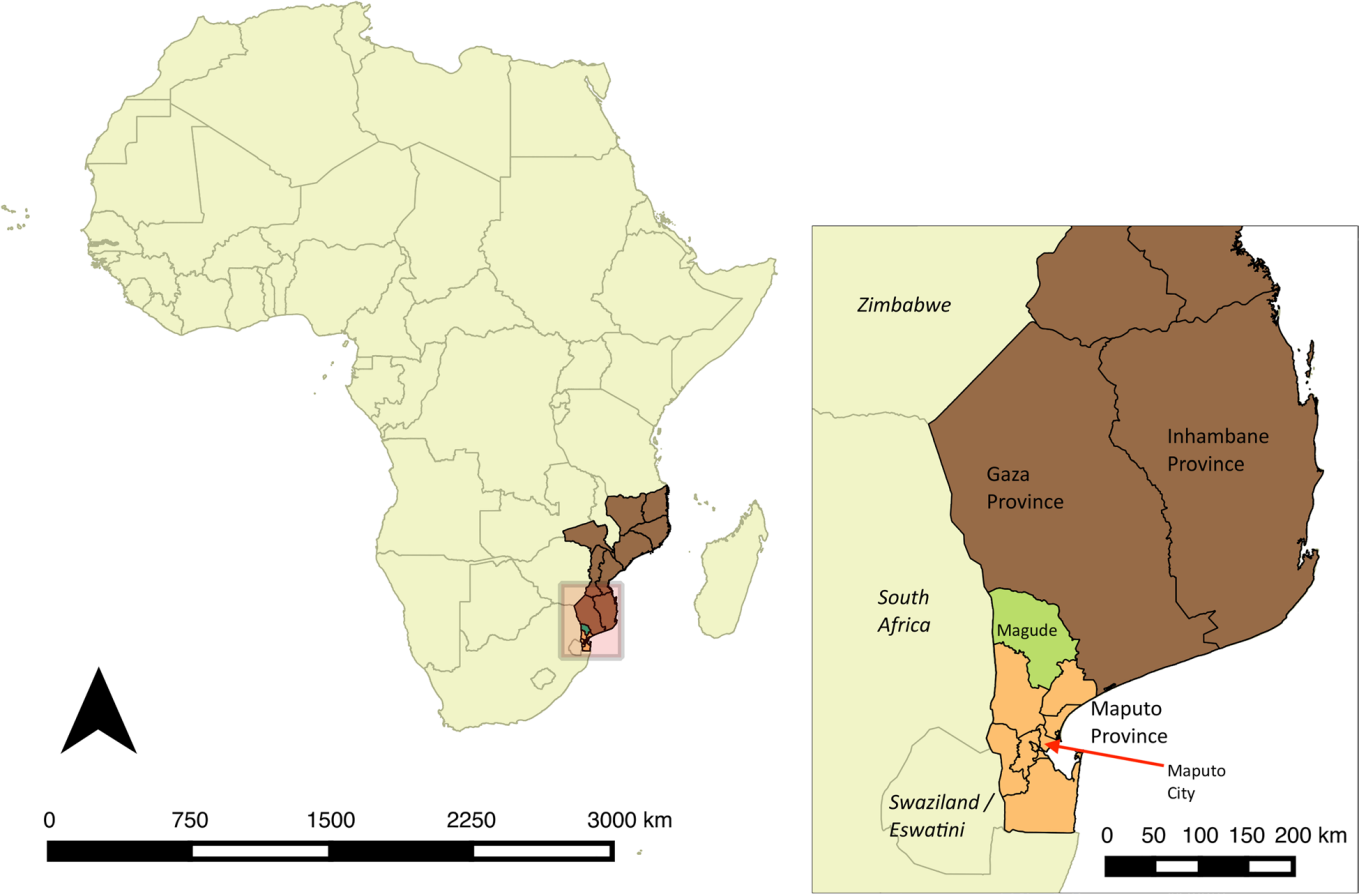
Map of southern Mozambique and districts of Maputo Province—Magude district (in green) and all other districts of Maputo province (in orange)

Mozambique has been one of the countries with the highest malaria burden in Africa and in the world, according to available data [1–3]. However, the Southern region of Mozambique (Fig. 1), especially Maputo City and Province, has historically implemented activities aimed at reducing the burden of malaria in the area, and consequently lower the number of importations into its neighbouring countries—South Africa and eSwatini (former Swaziland) [4]. The first indoor residual spraying (IRS) campaigns reported in southern Mozambique took place in 1946, using dichlorodiphenyltrichloroethane (DDT) and benzene hexachloride (BHC) [5, 6]. On the 27th of July of 1960, the World Health Organization (WHO) and the Portuguese government approved a malaria elimination plan designed by the Brazilian malariologist Dr. Ferreira for the three provinces south of the Save river—Maputo, Gaza and Inhambane—with a population of approximately 1.5 million individuals [7, 8]. This plan aimed to interrupt malaria transmission in the target areas and develop an elimination strategy with the neighbouring countries. To do so, a pilot project was implemented between 1960 and 1969 in the province of Maputo to test the feasibility to interrupt transmission in southern Mozambique through the use of IRS using DDT. The results from the pilot project indicated that malaria prevalence and vector density were significantly reduced to low and stable levels, and that ongoing transmission was mainly driven by the importation of cases from areas outside the pilot zone, and by outdoor biting [8], but the transmission was not interrupted.

The civil war (1977–1992) led to the interruption of IRS in the 70s and 80s, which only resumed after the war ended. During the 90s, IRS with lambda-cyhalothrin and deltamethrin was only performed in selected suburban areas of the country [4]. In the early 2000s, the Lubombo Spatial Development Initiative (LSDI) was initiated between the governments of Mozambique, eSwatini and South Africa, with the objective of developing the agriculture industries and economies of these countries while also attempting to significantly reduce malaria incidence in the bordering areas with South Africa and eSwatini [9]. Through LSDI, yearly rounds of household IRS with bendiocarb were conducted, a surveillance system was established, and availability of diagnostic and treatment was significantly enhanced between 2000 and 2011 in almost all districts of Maputo Province bordering eSwatini and South Africa (Table 1). The project failed to sustain the gains achieved, especially in Mozambique, due to financial constraints. However, the impact achieved on malaria incidence in South Africa and eSwatini led to the re-orientation of their respective malaria control programmes towards elimination [10].

**Table 1:** Main malaria control interventions deployed in Maputo Province since 1946 until 2014

Year	Area	Intervention	Source
1946–56	Semi-urban area of Maputo city and rural areas of the Limpopo Valley	IRS (DDT and BHC)	[5–7]
1960–69	Maputo Province	IRS (DDT)	[4]
1993	Suburban areas of most provincial capitals	IRS (deltamethrin and lambda-cyhalothrin)	
2000	IRS in Maputo province	2000–2011: “LSDI” IRS (bendiocarb) in all districts of Maputo province except Manhiça 2011–2017: District-level or targeted IRS in some districts of Maputo province 2017–2019: Province-level IRS through MOSASWA	[9, 10]
2003	Selected districts	IRS (with DDT, pyrethroids or bendiocarb)	[11, 39]
2005	Country-level	Introduction of RDTs	
2000	Country-level	ITNs for pregnant women and children under 5	
2005–2014	Provincial-level	Mass ITN distributions	
2017	Country-level	Universal distribution of LLINs^a^	
2005	Country-level	IPTp at ANC with SP	
2002–2004	Country-level	Introduction of AQ + SP as first line treatment	
2004–2009	Country-level	First-line treatment changed to AS + SP	
2009–2011	Country-level	First-line treatment changed to AL	
2011	Country-level	AQ + AS added as an alternative first-line treatment to AL for non-complicated malaria AS or parenteral QNN adopted for severe malaria treatment	

^a^Universal distribution of LLINS: one LLIN for every two people in the household

A significant reduction of malaria burden—measured as malaria admissions, parasite and spleen rates in children, or outpatient malaria incidence, depending on the year of evaluation—were reported after the implementation of the aforementioned IRS campaigns [4, 9]. However, it is worth noting that the evaluation of the impact on malaria incidence reported during LSDI may have been affected by the introduction in 2005 of the rapid diagnostic tests (RDTs) [11], which was a more specific diagnostic method than the previous one, based mostly on clinical symptoms. This, along with the introduction of a more efficacious first-line treatment (Table 1), may have significantly contributed to a decrease in the number of reported cases and deaths in the sprayed areas, considering that only approximately one third of children with fever reporting to a health facility in the area or detected in the community were positive for malaria infection [12, 13].

Alongside these efforts, since 2000 the NMCP of Mozambique intensified its control strategy through the implementation of the core interventions recommended by the World Health Organization (WHO) [14], i.e. efficacious anti-malarial drugs and vector control. Insecticide-treated nets (ITNs) began being distributed at the antenatal clinics and were also made available to children under 5 since the year 2000, and, in 2005, mass distribution of ITNs started in certain provinces of the country, aiming at 60% coverage of the most vulnerable populations, children aged less than 5 years and pregnant women. The country switched gradually from ITNs to long-lasting insecticide-treated nets (LLINs) starting in 2006. Since 2014, universal distribution of LLINs (one for every two individuals) has been conducted every 3rd year.

Around 2003, with the advent of the Global Fund to fight AIDS, Tuberculosis, and Malaria (GFATM), the NMCP reintroduced IRS with pyrethroids in selected districts across the country. In 2005, the MoH reintroduced DDT and expanded IRS implementation to over 40 districts countrywide through 2009, and to 62 districts between 2010 and 2015 [15].

Different artemisinin-based combinations have been used as a first-line treatment since 2002 in response to the growing resistance to chloroquine, and intermittent preventive treatment for pregnant women (IPTp) with sulfadoxine–pyrimethamine was introduced in 2005 at the antenatal clinics (Table 1). These interventions are funded by the Mozambican government with support mainly from the GFATM and the United States President’s Malaria Initiative (PMI), the latter since 2007 [11, 15].

**Fig. 2:**
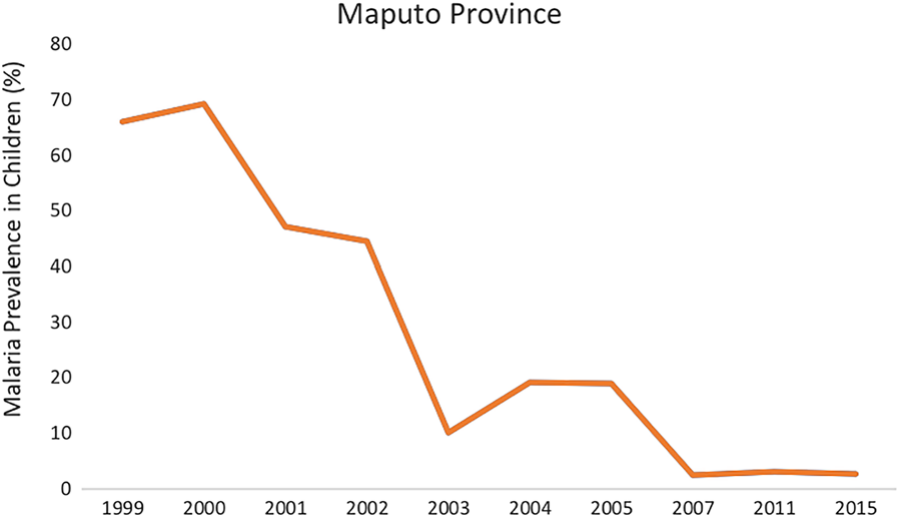
Historical malaria prevalence in children reported between 1999–2005 for 2–15 year olds (Sharp et al. [9]), and in 2007 (MIS [16]), 2011 (DHS [17]) and 2015 (MIS [18]) for < 5 year olds in Maputo Province, Southern Mozambique

The prevalence of malaria in children of Maputo Province has declined from > 60% before the year 2000, to < 30% by 2005, to < 3% in 2015 [9, 16–18] (Fig. 2). This reduction is probably multifactorial, associated with the significant socioeconomic changes that took place in the country during the last decades (expansion of urbanization, changes in land use, increased education, increased per capita GDP, etc.), as well as a result of the malaria control efforts, including LSDI, despite the relatively low coverage of IRS (< 50%) and ITNs (< 30%) estimated for Maputo province through national surveys conducted in 2007 [16], 2011 [17] and 2015 [18]. This reduction was not homogeneous within Maputo Province, as high incidence rates (> 200 cases per 1000 per year) were still observed in the district of Manhiça between 2000 and 2013 [19, 20]. The heterogeneity of transmission is also mirrored at country level, where estimates of *Plasmodium falciparum* malaria prevalence ranged from 16% in Gaza province (north of Maputo) to 67.6% in the northern province of Zambezia [18], and the WHO estimated that approximately 9.5 million cases and 16,000 deaths were due to malaria in 2015 [3].

## The rationale for malaria elimination in southern Mozambique

While the last decade had witnessed significant reductions in the burden of malaria throughout the country, the gains have since stalled, and an increase in disease incidence consistent with modelled estimates [21, 22] has been reported since 2014 throughout the country [23]. In this context, it was clear that business as usual was no longer an option for Mozambique, and the country had no option but to intensify its control efforts to meet the targeted decrease in malaria incidence and mortality in the national strategic plan [11]. The NMCP of Mozambique followed the recommendations established by the WHO of increasing the coverage of all core interventions (vector control, case management, and case surveillance) throughout the country, while simultaneously developing and implementing a strategic plan to accelerate to elimination in the south, where malaria burden was the lowest [24].

While recognizing that malaria elimination in Mozambique is extremely ambitious, there were multiple reasons that encouraged the country to embark on an elimination effort in the south in 2014. First, there was a need to demonstrate the feasibility and impact of malaria elimination to inform the design of new elimination strategies in endemic countries where scale-up of control is still challenging, and the question remains as to whether elimination is really feasible and biologically plausible [22, 25, 26]. Multiple research efforts were already underway across varying geopolitical and transmission zones to inform global policies for elimination, the majority in countries with low to moderate transmission levels nationally, such as South Africa, eSwatini [10] and Namibia [27], or with strong health systems, as countries in the Asia–Pacific region [28]. Mozambique, as well as Zambia, where intensive malaria control projects were already ongoing [29], provided an optimal environment to evaluate whether interventions aiming to interrupt malaria transmission could be implemented, and elimination achieved and sustained cost-effectively. Proving that malaria elimination could be achieved in these countries would provide a convincing advocacy and mobilization argument for aiming for elimination in Africa. It would additionally offer a good counter-argument to the discouraging predictions of future malaria increases world-wide if no acceleration efforts were put in place [21, 30].

Second, the opportunity for regional impact was higher than ever. Surveillance data from eSwatini and South Africa reportedly identified imported cases from Mozambique as one of the main challenges preventing them from completely interrupting transmission. As a result, there was a growing recognition that successful elimination in South Africa and eSwatini would require a cross-border approach that aggressively targets southern Mozambique, the major source of malaria importation in the region [10].

Finally, an unprecedented enthusiasm and support from local and global partners and funding institutions arose to define and implement a malaria elimination strategy for the south of Mozambique. This enthusiasm needed to be channelled through the creation of a coordination mechanism that would facilitate partner collaboration and provide a structure through which the NMCP could lead all partners under a single plan. It would also serve to identify the human resources, management infrastructure and health systems gaps required to embark on a malaria elimination mission led nationally as recommended by WHO [31].

## Setting the scene for malaria elimination in Southern Mozambique

Responding to the growing needs to address all aspects that positioned southern Mozambique in the path of malaria elimination, in 2014 the La Caixa Foundation (LCF) and the Bill & Melinda Gates Foundation (BMGF) jointly funded a grant to the Barcelona Institute for Global Health (ISGlobal) and the *Centro de Investigação em Saúde de Manhiça* (CISM) to design and implement, in collaboration with the Mozambican NMCP, a 5-year malaria elimination programme with the overall goal of designing, implementing and evaluating a malaria elimination strategy for southern Mozambique. Around this time, the Elimination 8 Initiative and the Mozambique, South Africa and eSwatini (MOSASWA) regional initiative were created through GFATM funding, with the main objective of significantly reducing malaria sub-nationally in Southern Mozambique in order to achieve the malaria elimination target in South Africa and eSwatini by 2020 [32].

The 5-year malaria elimination program that resulted from the LCF and the BMGF partnership, spearheaded the introduction of the malaria elimination strategy into the country’s agenda. The program aimed to learn from the previous malaria elimination attempts in Africa by adopting a horizontal approach that focused on (i) promoting national ownership and partner coordination to work towards the goal of malaria elimination in local and regional initiatives, (ii) generating epidemiological and entomological intelligence through strengthened surveillance systems to inform the deployment of malaria control strategies and evaluate their impact; and (iii) evaluating the impact and feasibility of interventions aimed to interrupt malaria transmission through a demonstration project of malaria elimination in Magude, a district of Maputo Province.

## The creation of a national alliance for malaria elimination

Acknowledging the importance of national ownership for the success of any public health activity, the malaria elimination programme placed substantial efforts on the creation of a national platform to support the NMCP to design, fund and implement a national malaria elimination plan for the south. As a result, the Mozambican Alliance Towards the Elimination of Malaria (MALTEM) or “*Aliança pela Eliminação de Malária em Moçambique*” (ALEMMO) in Portuguese, was created as a collaboration between independent institutions working on malaria in Mozambique. Chaired by the NMCP, MALTEM was launched in July 2015 and included members from various sectors: Multilateral Agencies (WHO, Roll Back Malaria Partnership, United Nations Children’s Fund (UNICEF) and GFATM); bilateral agencies (PMI and United States Agency for International Development (USAID)); academic and research centres (CISM and ISGlobal); and private foundations (Good Bye Malaria (GBM), Fundação para o Desenvolvimento da Comunidade (FDC), BMGF, LCF, the Clinton Health Access Initiative (CHAI), Malaria Consortium and World Vision).

MALTEM’s main objectives stated in its Terms of Reference were to “*create the necessary knowledge to inform an operational elimination plan and roadmap for malaria elimination in Mozambique; ensure that the NMCP has the necessary capacities to implement innovative strategies to improve control of malaria and interrupt transmission; align efforts for political engagement and raise further resources of funding including domestic ones; and identify synergies to ensure that potential overlaps or duplicated efforts are avoided and that the best uses of resources are guaranteed*.” In-country members of MALTEM met regularly upon NMCP’s request and once a year with the MoH and its funders (BMGF and LCF).

An Advisory Committee for MALTEM (MAC) including malaria elimination experts and NMCP managers from Africa was constituted to provide independent scientific and strategic advice to MALTEM and support the evidence generation process to achieve malaria elimination in Southern Mozambique. MAC members met on an annual basis between 2015 and 2018. Finally, an independent national Malaria Technical Advisory Committee (MTAC) was created under a ministerial decree, to provide technical and scientific advice to the MoH to develop evidence-based policies for the control and eventual elimination of malaria in the country. This body contributed to provide a consensual and stable environment and robust leadership to strengthen the malaria control activities in Mozambique inspired on the successful model of the WHO Malaria Policy Advisory Committee (MPAC).

As a result of the creation of these platforms, the 2017–2022 National Strategic Plan of the Mozambican NMCP included the goal of malaria elimination and created a specific Technical Working Group (TWG), opening the doors to the development and implementation of a detailed subnational elimination plan for the southern, lowest endemic districts. An evaluation was conducted early in 2015 to identify the human resource needed to strengthen the central NMCP in order to respond to the demands posed by the elimination agenda. This analysis revealed that while most NMCP positions were already filled, the existing NMCP personnel lacked necessary core capabilities and competencies, including programme management, concept development, analytical skills and strategic planning, coaching and training skills. In addition to sharing the findings of this assessment with key NMCP stakeholders, three technical staff were hired by MALTEM and seconded to the NMCP to fill the most relevant gaps identified at the time, namely, one vector control officer at central level, one entomological assistant at provincial level (for Maputo province) and one surveillance officer at district level in Magude.

Moreover, in order to address some of the gaps identified in terms of core competencies and capabilities of the NMCP personnel, several MALTEM members organized short courses tailored to individuals at national, provincial, district and community level between 2015 and 2018 on a variety of topics relevant to malaria control and elimination. These courses included malaria surveillance and entomological training (by CISM and ISGlobal), as well as a variety of community-level training on IRS deployment (by Good Bye Malaria), community engagement (by CISM and FDC), and mass or focal drug administration (MDA) activities (by CISM and ISGlobal). Every year, since 2016, CISM and ISGlobal supported the participation of NMCP personnel at the Science of Eradication courses, organized by the Universities of Barcelona, Harvard, and Basel. Additionally, a large group of young Mozambicans was hired to take part in the research activities at CISM to acquire programme implementation and operational research experience, with the vision that they would maintain and expand the in-country expertise on malaria surveillance, epidemiology and entomology.

Several advocacy events were organized involving various Mozambican leaders at all levels. These included briefings with community leaders, district/province health and administrative leaders, as well as the Minister of Health. These meetings aimed to socialize the idea of elimination and establish an inclusive decision-making process to ensure national ownership at all levels. The advocacy effort was also aimed at raising domestic financial commitments both from the private sector as well as from the government. While political commitment was achieved at all levels, leveraging national resources for malaria elimination was challenged by the massive financial crisis that the country is experiencing since 2015.

### Strengthening epidemiological and entomological surveillance systems

In 2015, the Mozambique Health Information System changed the procedures for collecting malaria data from the district to the national level, from a paper-based system to an electronic system using the District Health Information System 2 (DHIS2) platform to obtain monthly malaria indicators from all districts in the country, with support from the GFATM. Aggregate malaria data are reported by age group (below and above 5 years of age), including total outpatient visits, RDTs and/or microscopy performed, positive RDTs/microscopy, suspected malaria cases (if not tested for any reason but assumed to be malaria according to symptoms) and treatment provided (ACT). However, data from health facilities (HF) and community health workers (CHW) are still collected on paper and sent to the district to be entered electronically into DHIS2. Since 2015, the malaria elimination programme further expanded the DHIS2 system to obtain monthly electronic data from all HFs and CHWs in four of the eight districts of Maputo Province, namely Marracuene, Moamba, Manhiça, and Magude; while also establishing a rapid reporting system for weekly data collection in the district of Magude. Quarterly data quality audits were gradually established in the 4 districts to evaluate the timeliness, completeness, and accuracy of the data collected.

An entomological surveillance platform was established at six sentinel sites in Magude district in 2015 and in one additional sentinel site in Xinavane town in 2016 to (i) better tailor vector control strategies to the entomological context of the area and (ii) assess the effectiveness of vector control interventions. Vector species composition, mosquito densities and infection rates were monitored in/around Magude town, and insecticide resistance was monitored annually to inform the selection of IRS insecticides. Monthly residual efficacy of IRS was also monitored.

Based on lessons learned, entomological surveillance activities were redesigned in 2018 and new entomological surveillance techniques were implemented, such as human-baited tent traps placed both indoors and outdoors, early-morning pyrethrum spray catches, and window exit traps. Activities were also expanded to Gaza and Inhambane provinces in order to collect essential, timely and quality entomological information to monitor the vector population and inform the IRS strategy planned for Gaza and Inhambane under the MOSASWA regional initiative.

### The “Magude” project: assessing the feasibility of malaria elimination

Despite the successes in controlling the disease in sub-Saharan Africa, the region still houses the countries with the highest malaria burden in the world [33]. Interrupting malaria transmission and, ultimately, eliminating the parasite from this region is a long-term goal that will require innovation through research and deployment of elimination strategies specifically tailored to high burden areas [34]. This was precisely the goal of the Garki Project, undertaken in Northern Nigeria in the context of the Global Malaria Eradication Program (GMEP) between 1969 and 1976, to assess the feasibility of interrupting transmission in the African savanna with the tools available at the time. This project compared 7 rounds of IRS with propoxur, to the same IRS approach in combination with 9 or 23 rounds of MDA using sulfalene-pyrimethamine throughout a 2-year period. The main conclusion from the Garki project was that malaria burden had been significantly reduced through these strategies, but elimination was not achieved during the established intervention period with the interventions deployed, and malaria rebounded after its discontinuation [35].

The results have often been interpreted as a confirmation that the goal was at that point unachievable. However, today, newly available tools, together with innovative strategies, may facilitate achievement of malaria elimination in the low transmission areas of Africa [14]. In line with the renewed interest in malaria elimination in southern Mozambique, a malaria elimination project called the *“Magude project*” was designed by ISGlobal, CISM and the NMCP to revisit the feasibility of malaria elimination in endemic countries of Africa with the currently available tools and technologies.

The district of Magude (Maputo province, southern Mozambique) was selected as the area where the feasibility of malaria elimination would be evaluated based on a series of district characteristics that would pose the type of challenges expected to be faced by the NMCP while implementing a malaria elimination campaign country-wide. This district was included in the baseline malaria surveys conducted in preparation for the 1960s elimination plan, which revealed a 44% prevalence of infection by microscopy in 1958 [7]. It also received the LSDI activities (Zone 3), during which the prevalence by RDT dropped from 77% in 2003 to 33% in 2005 [9] and to < 10% between 2008 and 2011 [36]. The number of cases reported per year from Magude after the end of LSDI increased from 9845 in 2012 to 13,661 in 2014 (38% increase). However, this increasing trend was generally observed throughout the country, but no comparisons could be made with regards to incidence trends during the LSDI due to the inaccuracy of the national routine surveillance data [3, 36].

The aim of the *Magude project* was to assess the feasibility and impact of a comprehensive malaria elimination package that combined routine malaria control activities with innovative interventions to interrupt transmission. The package of interventions consisted of: (i) standard of care using HRP2-based RDTs for diagnosis and artemether–lumefantrine for treatment, delivered by the MoH in the district; (ii) enhanced entomological and epidemiological intelligence through an improved surveillance and reporting system; (iii) a strong community engagement campaign to maximize acceptance and coverage of interventions; (iv) universal coverage of IRS with DDT and/or Actellic 300 CS (pirimiphos-methyl) performed at the end of the dry season on top of the LLINs distributed by the NMCP in 2014 and 2017; and (iv) two population-wide Mass Drug Administration (MDA) rounds with dihydroartemisinin–piperaquine (DHAp) per year for two consecutive years followed by reactive focal Mass Drug Administration (rfMDA) of contacts of passively detected cases. Several research studies also took place simultaneously in order to assess the clinical and socio-demographic profile of cases with malaria infection through time; the community’s adherence and acceptability to the interventions; DHAp safety and resistance; HRP2 deletions; LLIN integrity and bio-efficacy, the sleeping behaviours in the community in relation to LLIN use; the cost-effectiveness of the interventions, and their impact on school and work absenteeism.

The rationale was that a combination of vector control and population-wide anti-malarial drug interventions would lead to a significant reduction in the mosquito population, as well as in the human parasite reservoir. In the context of a diminished vector population, the long prophylactic effect of repeated anti-malarial drug doses would protect individuals from pre-treatment infected mosquitoes, while the new generation of mosquitoes would feed onto post-treatment non-infected humans thus leading to the interruption of the malaria transmission cycle.

**Fig. 3:**
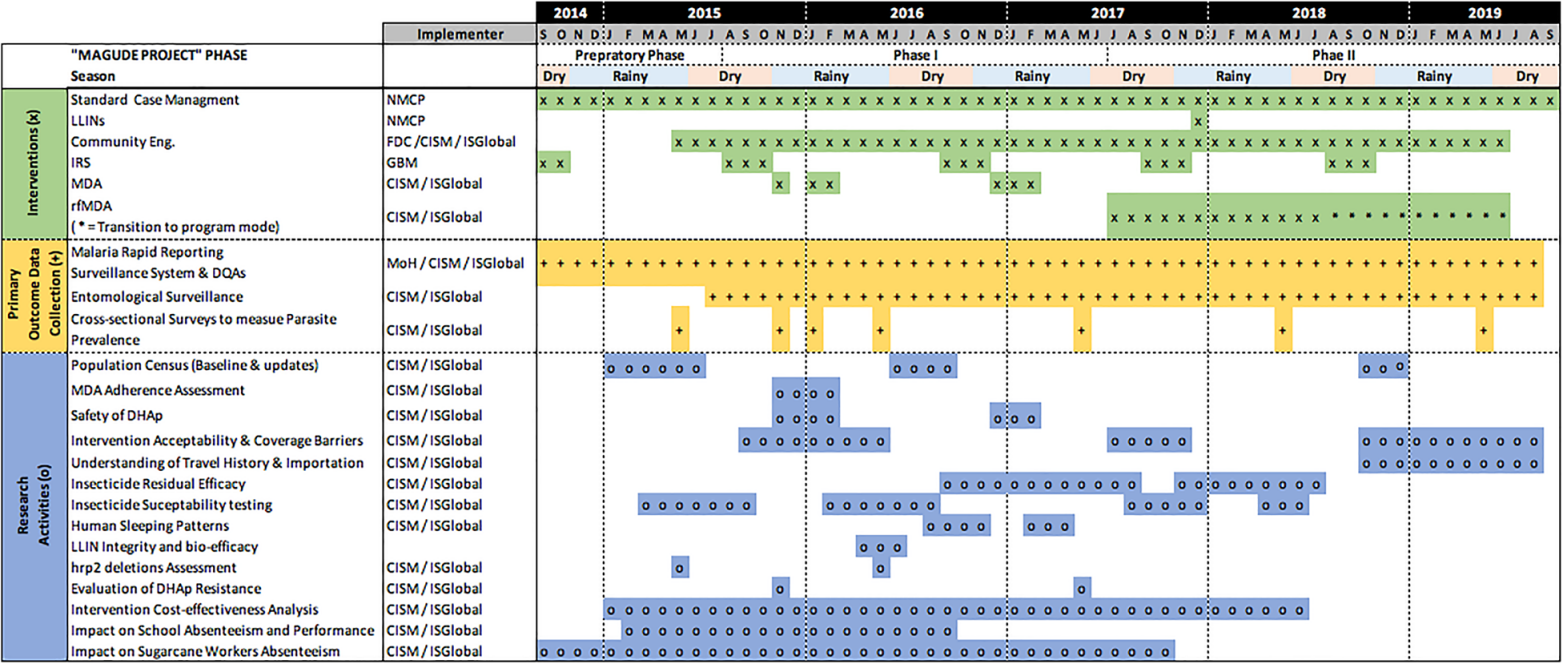
Design of the *Magude Project* including the interventions implemented (green, “x”), the activities for the collection of primary data to evaluate the impact of the interventions (yellow, “+”), and the research activities performed during the project (blue, “o”) between 2014 and 2019

The project was divided into three phases presented in Fig. 3 in detail:

1. *Preparatory phase* (*September 2014 to August 2015*) a census and a malaria infection prevalence survey were conducted to obtain community baseline data. Epidemiological and entomological surveillance systems were established. During this phase, two studies were performed in the neighbouring district of Manhiça to evaluate the efficacy of chloroquine and the prevalence of G6PD deficiency for the use of primaquine, to inform about their potential use in future elimination interventions [37, 38].
2. *Phase I* (*August 2015 to June 2017*) implementation of the first set of interventions aiming at interrupting transmission. One round of IRS followed by two rounds of MDA were implemented during two consecutive rainy seasons. A community engagement campaign was also conducted to increase the use of LLINs and maximize acceptance of IRS and MDA. The census was updated in 2016 and two more malaria infection prevalence surveys were conducted at the end of each transmission season (May 2016 and May 2017).
3. *Phase II and transition to programmatic mode* (*July 2017 to September 2019*) implementation of a second set of interventions aiming at sustaining the gains achieved during phase I through the deployment of three more annual rounds of IRS at the end of the dry season of 2017, 2018 and 2019, coupled with rfMDA established in July 2017; a universal LLIN distribution conducted by the NMCP in December of 2017; two parasite surveys in May of 2018 and 2019; and another census update at the end of 2018.

## Lessons learned

Several lessons were learnt from the implementation of the malaria elimination programme. Bringing several NMCP stakeholders together to form MALTEM was not an easy undertaking for several reasons. First, there was a strong scepticism about the feasibility of elimination in such a high malaria burden country; second, most stakeholders’ institutions had not formally endorsed the elimination agenda; third, the priority for the NMCP and its partners were the high burden regions of the country, particularly northern Mozambique. These constraints hampered the functioning of MALTEM, limiting its efficiency. On the other hand, other drivers were key in fuelling the alliance, particularly WHO’s headquarters’ strong leadership, through the endorsement of the global technical strategy by the 2015 World Health Assembly and BMGF’s leadership, especially in the field of evidence generation across the globe.

A key lesson learned from MALTEM’s advocacy process is that advocacy for elimination has to begin by targeting the country’s leadership at its highest level, in order to elicit not only their political support but also a financial commitment from domestic funding.

The introduction of malaria elimination in the NMCP’s agenda was another challenge, not only for the reasons listed above but most importantly due to the risk of political fallout that could arise from prioritizing elimination activities in one region over other competing needs. To overcome this, rather than focusing on elimination in the south, the NMCP strategy presented a more dynamic approach that would be guided by local epidemiological contexts. Another key factor that facilitated the adoption of the elimination agenda was the country’s regional commitment with its southern neighbours in the context of MOSASWA and E8. While the NMCP eventually embraced the elimination agenda and included it in its strategic plan, the NMCP’s investments priorities naturally remained in northern Mozambique, subjecting the implementation of elimination-oriented activities to the availability of specific additional funding.

In terms of strengthening the surveillance system the authors had initially thought of a hybrid approach; on one hand, to support the DHIS2 surveillance platform implementation in the district, led by the MoH; whereas, on the other hand, to invest in a parallel surveillance system, building on the long-term experience of running a round-the-clock facility-based passive case detection at Manhiça district hospital. The latter, however, proved to be a colossal task given the level of investment that would need to be made in terms of personnel, having prevailed the former. This, however, came with its own human, logistical and technological challenges that had to be overcome through direct provision of technical, logistical and technological support. In order to minimize the risk of disruption of the surveillance after the end of the project, no changes were made to the reporting tools and the paper-based reporting system remained as a backup system throughout the project. Moreover, the project team worked tirelessly to attract new funding to continue supporting surveillance while continuing to advocate for its full absorption by the health system itself.

The key lessons from this experience in Magude in relation to surveillance were that any similar initiative should avoid trying to setup parallel systems as these can be costly and not sustainable in the long run. Also, while, technical support is important, it is equally important to plan for some level of logistic support while continuously advocating for the health system to fully absorb the surveillance needs.

Overall, the Magude project faced several challenges inherent to the implementation of MDA in a research context, using a relatively new drug for which there was still limited safety data especially in individuals without the disease. To overcome this, high-level advocacy had to be undertaken at all levels, from central, provincial to district level prior to the community mobilization. In addition, formative research in social science was undertaken before, during and after the MDAs to continuously inform the deployment of the MDA intervention and adapt it according to the learnings being acquired.

## Concluding remarks

Southern Mozambique has experienced several malaria elimination attempts during the twentieth century, which together with the NMCP’s efforts to control the disease nationally and the socio-economic development, have led to a significant reduction of malaria prevalence in the area. In 2014, a partnership between the La Caixa Foundation and the Bill and Melinda Gates Foundation prompted the creation of a malaria elimination programme in southern Mozambique that aimed to pave the way for malaria elimination in the country through the establishment of nationally-led platforms to design a plan for the south, based on the evidence generated through a malaria elimination demonstration project in the district of Magude. The Mozambican Alliance Towards the Elimination of Malaria (MALTEM) and the Malaria Technical Advisory Committee (MTAC) were created to strengthen partner communication, facilitate the adoption of evidence-based policies and secure funding for malaria elimination. As a result, the goal of malaria elimination in areas of low transmission intensity was included in the national malaria strategic plan for 2017–22 and several partners of the NMCP have started to work towards this goal. Additionally, the partnership led to the design, implementation and evaluation of the *Magude Project*, for the generation of in-country evidence on the feasibility and affordability of malaria elimination in the south to guide policymaking. The implementation of all these activities has offered key lessons that should be considered in any future malaria elimination endeavours in Mozambique, which might also be applicable in other countries aiming for elimination. Overall, since 2014, the Mozambican NMCP has significantly strengthened its approach to malaria policy-making, with a focus on elimination, through the creation of coordination mechanisms that offer their technical and financial support, as well as through the generation of in-country evidence to guide malaria elimination strategies in Mozambique.

## Data Availability

All data presented in this article have been previously published. Sources of data can be found in the reference list.

## Abbreviations

ACT: artemisinin-based combination therapy
ALEMMO: Aliança pela Eliminação de Malária em Moçambique
BHC: benzene hexachloride
BMGF: Bill and Melinda Gates Foundation
CHAI: Clinton Health Access Initiative
CHW: community health workers
CISM: Centro de Investigação em Saúde de Manhiça
DDT: dichlorodiphenyltrichloroethane
DHAp: dihydroartemisinin–piperaquine
DHIS2: District Health Information System 2
FDC: Fundação para o Desenvolvimento da Comunidade
GFATM: Global Fund to fight AIDS, Tuberculosis and Malaria
GMEP: Global Malaria Eradication Program
IRS: indoor residual spraying
IPTp: intermittent preventive treatment for pregnant women
ISGlobal: Barcelona Institute for Global Health
ITNs: insecticide-treated nets
LCF: La Caixa Foundation
LLINs: long-lasting insecticide-treated nets
LSDI: Lubombo Spatial Development Initiative
MALTEM: Mozambican Alliance Towards the Elimination of Malaria
MDA: Mass Drug Administration
MIS: Malaria Indicator Survey
MoH: Ministry of Health
MPAC: Malaria Policy Advisory Committee
MTAC: Malaria Technical and Advisory Committee
NMCP: National Malaria Control Program
PMI: President’s Malaria Initiative
RDTs: rapid diagnostic tests
rfMDA: reactive focal Mass Drug Administration
TWG: Technical Working Group
UNICEF: United Nations Children’s Fund
WHO: World Health Organization
